# Recent insights of fucoidan probiotic and its effect on gut microbiota

**DOI:** 10.17179/excli2023-6219

**Published:** 2023-06-22

**Authors:** Bassma H. Elwakil, Moaaz T. Hamed, Keshav Raj Paudel

**Affiliations:** 1 Department of Medical Laboratory Technology, Faculty of Applied Health Sciences Technology, Pharos University in Alexandria, Alexandria, Egypt; 2 Department of Botany & Microbiology, Faculty of Science, Alexandria University, Alexandria 21568, Egypt; 3 Department of Oriental Medicine Resources, Mokpo National University, Muan-gun, Jeonnam 58554, Republic of Korea

## ⁯

Fucoidan is a fucose-based, sulfated polysaccharide found in sea cucumbers and brown algae (Fitton et al., 2019[4]). Fucoidan was reported to have several biological activities, such as anti-inflammatory, antithrombotic, anticoagulant, antibacterial, antiviral, antioxidant, anticancer, antifibrotic, and immunomodulatory (Charboneau et al., 2018[1]). Because of their antibacterial properties, they were considered excellent candidates for several applications. Fucoidan was used as a natural and "Generally Recognized as Safe" (GRAS) health product or as an antibacterial agent to suppress *Helicobacter pylori* (Chua et al., 2015[2]). 

Fucoidan's content and structure vary depending on cucumbers and brown algae species, the sampling location, harvesting season, and the applied extraction methods (Mak et al., 2013[7]). Fucoidans are commonly recovered from various natural resources using water, acid bath, or microwave heating. The extraction procedure affects the fucoidan's bioactivity while the sample source affects the molecular weight (Mw) which may range between 10,000 and 100,000 Da (Fitton et al., 2019[4], Wang et al., 2019[9]).

Fucoidan extracts have recently received regulatory approvals for meals and dietary supplements usage in several countries around the world. The Australian producer Marinova registered fucoidan extracts from *Undaria pinnatifida* and *Fucus vesiculosus* as "Generally Recognized as Safe" (GRAS) with the US Food and Drug Administration. The FDA had no more issues with the two GRAS findings, which allowed the oral consumption of up to 250 mg/day GRAS fucoidan extracts (Fitton et al., 2019[4]). In the European Union, the GRAS fucoidan extracts were appraised by the European Commission and determined as markedly correspondent to the original seaweeds from which they were extracted; consequently, they were approved as novel foods under Commission Implementing Regulation (EU) 2017/2470, for consumption of up to 250 mg/day (Fitton et al., 2019[4]). The corresponding regulators in Canada and Australia have authorized a variety of fucoidan extract-containing drugs. In Australia, fucoidans have been licenced for *Undaria pinnatifida* and *Fucus vesiculosus* in a species-specific context. They were recognized as enumerable constituents of their botanical parent (seaweed) (Fitton et al., 2019[4]).

On the other hand, probiotics may produce antimicrobial substances, modulate the immune system and gut microbiota, enhance nutrient absorption, and providing the host with health advantages. Recently, probiotics usage has garnered considerable interest in healthcare due to their safety and beneficial effect on the host's health (Clemente et al., 2018[3]). The most commonly used probiotics are lactic acid bacteria, such as *Lactobacillus* spp. and *Pediococcus* spp. (Clemente et al., 2018[3]). For example, *Lactobacillus rhamnosus* (*L. rhamnosus*) is extensively distributed throughout the human body and has an important effect on human health. *L. rhamnosus* was reported to boost short-chain fatty acid (SCFA) synthesis in the stomach which can control pH, suppress pathogenic microbes, fight for attaching sites, and exert antimicrobial and anti-inflammatory actions (Markowiak-Kopec and Slizewska, 2020[8]). 

Fucoidan has a prebiotic effect on the development of probiotics at optimal concentrations, according to Zhu et al. (2021[10]). They also reported that the media pH values of all tested samples decreased during the first eight hours of incubation with fucoidan, indicating that probiotics had a slow metabolic rate. From the eighth to the sixteenth hour, the significantly decreased pH of *L. rhamnosus*, suggesting that the development and metabolism of probiotics were greatly accelerated, producing an abundance of organic acids. This data implies that the greatest dosage of fucoidan hindered the probiotics' metabolic capacity (Zhu et al., 2021[10]). Another important effect of fucoidan consumption relies on endotoxin detoxification. Fucoidan administered subcutaneously or orally was highly protective in a mouse model infected with endotoxin (Kuznetsova et al., 2014[6]), which may pave the way for using fucoidan as an extremely desirable dietary supplement.

Fucoidans have been shown to induce favorable microbiota alterations (Chua et al., 2015[2]). Fucoidans can regulate the microbiota and inhibit the binding of pathogenic microorganisms. By blocking the interaction of mucins with the secreted TpsA/CdiA (a virulence factor) and increasing the establishment of the useful *Bacteroides* population, marine prebiotic fucoidans may facilitate an enhanced recovery from dysbiosis and *P. aeruginosa* decolonization from the gut (Janapatla et al., 2023[5]).

Fucoidan was shown to control probiotics proliferation, metabolism, shape, and antibacterial activity. Fucoidan may therefore act as a probiotics' modulator or a prebiotic of marine-origin, providing new vision into the prebiotic possible bio-applications such as promoting tissue repair and regeneration, preventing tissue infections, and enhancing the oral normal flora as well as the gastrointestinal, and vaginal floras.

## Disclosure of financial and competing interests

The authors have no relevant affiliations or financial involvement with any organization or entity with a financial interest in or financial conflict with the subject matter or materials discussed in the manuscript. This includes employment, consultancies, honoraria, stock ownership or options, expert testimony, grants or patents received or pending, or royalties.

No writing assistance was utilized in the production of this manuscript.
